# The Medical Journal Is the True Post-Graduate Instructor

**Published:** 1896-04

**Authors:** 


**The Medical Journal is the True Post-Graduate Instructor.**—The *Archives of Pediatrics* for February contains the following remarks regarding modern medical journalism: They are a part of an unsigned editorial that is, as we believe, from the incisive pen of Dr. Floyd M. Crandall, and they are a part of a series of editorials on the building up of a complete professional character. He says: "Three avenues are open to the physician by which he may keep abreast of the times—books, medical journals and post-graduate schools. The last of these are accessible to but few, and present instruction adapted only to certain cases. The last editions of standard medical books are indispensable to the true student. In the text-book and monograph he finds the various subjects of medical interest reviewed systematically and discussed in all their details. In the medical journal, however, he finds the record of medical progress as he can find it in no other place. The journal with its various departments admits of all classes of medical literature, and contains many practical and important articles which would not be adapted to the more pretentious and dignified bound volume. The well arranged journal covers the whole field of medical writing, from the scientific original article to the clinical report, which not infrequently furnishes a key to some puzzling case. Such assistance in a single case often proves of a value not to be estimated in dollars and cents. Nearly all the best papers that appear in medical journals are first read in societies. The discussions which they elicit are frequently of even more practical value than the papers themselves. Society reports are justly regarded, therefore, as of very great value to the practitioner. A department of book reviews, when conscientiously conducted, is also a factor of value to the medical man. The average doctor is limited in the number of books he is able to purchase. A discriminating review, which does not fear to criticise, but does not criticise for the mere sake of criticising, enables him to judge both of the scope of a book and its probable value to him. He is thus able to place in his library those books from which he will derive the most good in his own particular class of practice."

